# Amyloid deposits and inflammatory infiltrates in sporadic inclusion body myositis: the inflammatory egg comes before the degenerative chicken

**DOI:** 10.1007/s00401-015-1384-5

**Published:** 2015-01-13

**Authors:** Olivier Benveniste, Werner Stenzel, David Hilton-Jones, Marco Sandri, Olivier Boyer, Baziel G. M. van Engelen

**Affiliations:** 1 Département de Médecine Interne et Immunologie Clinique, Assistance Publique-Hôpitaux de Paris, GH Pitié-Salpêtrière, Université Pierre et Marie Curie, Inserm, U974, DHU I2B Paris, France; 2 Centre de Référence des Pathologies Neuromusculaires Paris Est, Institut de Myologie, Paris, France; 3 Department of Neuropathology, Charité-Universitätsmedizin, Berlin, Germany; 4 Department of Neurology, West Wing, John Radcliffe Hospital, Oxford, UK; 5 Department of Biomedical Science, Venetian Institute of Molecular Medicine, University of Padova, Padua, Italy; 6 Inserm, U905, Rouen, France; 7 University of Rouen, IRIB, Rouen, France; 8 Department of Neurology, Radboud University Medical Center, Reinier Postlaan 4, Nijmegen, The Netherlands

**Keywords:** Inclusion body myositis, Pathogenesis, Immune responses, Autoimmunity, Amyloid deposits

## Abstract

Sporadic inclusion body myositis (sIBM) is the most frequently acquired myopathy in patients over 50 years of age. It is imperative that neurologists and rheumatologists recognize this disorder which may, through clinical and pathological similarities, mimic other myopathies, especially polymyositis. Whereas polymyositis responds to immunosuppressant drug therapy, sIBM responds poorly, if at all. Controversy reigns as to whether sIBM is primarily an inflammatory or a degenerative myopathy, the distinction being vitally important in terms of directing research for effective specific therapies. We review here the pros and the cons for the respective hypotheses. A possible scenario, which our experience leads us to favour, is that sIBM may start with inflammation within muscle. The rush of leukocytes attracted by chemokines and cytokines may induce fibre injury and HLA-I overexpression. If the protein degradation systems are overloaded (possibly due to genetic predisposition, particular HLA-I subtypes or ageing), amyloid and other protein deposits may appear within muscle fibres, reinforcing the myopathic process in a vicious circle.

## Introduction

Sporadic inclusion body myositis (sIBM) is the most frequent acquired progressive myopathy presenting over 50 years of age in Western populations [13], albeit rare. For example, a recently published Dutch study from a tertiary referral centre identified only 64 patients from 7 specialized neuromuscular centres in a country with 16 million inhabitants [26], giving an estimated prevalence of 4.9 patients per million inhabitants in The Netherlands [13]. This may be an underestimate since sIBM can mimic other forms of myositis including polymyositis, hereditary myopathies of the limb girdle phenotype (see below) and the lower motor neuron variant of ALS. sIBM is a disabling (but not in itself lethal) muscle disease with first symptoms typically presenting in the 6th decade of life [17]. The natural history of the condition is one of the relentless progression of weakness, with an estimated loss of strength between 3.5 and 5.5 % per year [17, 26]. Pneumonia, secondary to immobility, respiratory muscle weakness and aspiration due to dysphagia, is a common terminal event [17, 26].

**Fig. 1:**
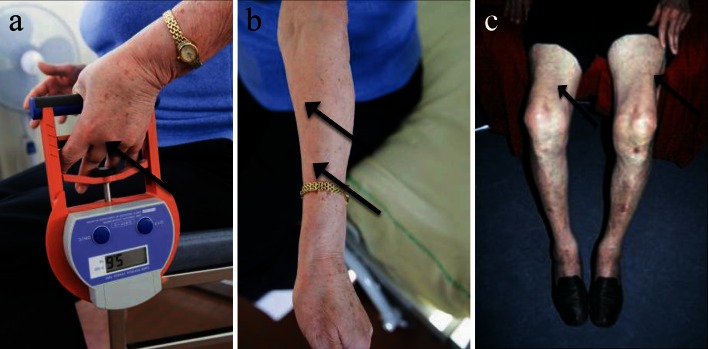
Clinical features of sIBM. **a** Finger flexors weakness (*arrow* maximum obtained by this patient: 5.6 kg, normal >21 kg for a woman at 70 years old), and **b** atrophy (*arrows*). **c** Quadriceps atrophy (*arrows*) for a man at 75 years old

There is a highly characteristic pattern of limb muscle involvement with selective weakness of finger flexion (Fig. 1a, b) and knee extension (Fig. 1c). This pattern was noted by early investigators but considered of secondary importance to pathological features in establishing the diagnosis [42]. More recently, it has been proposed that clinical features are paramount and may allow the diagnosis of sIBM in the absence of what had previously been considered to be canonical pathological features (Table 1) [19, 82]. Such criteria were assessed prospectively in a large cohort of IBM (*n* = 200) and non-IBM patients with other neuromuscular disorders (*n* = 171) [59]. They are highly specific (range 98–100 %) with a sensitivity of 77–84 % for probable IBM depending on the criteria used [19, 82]. Furthermore in this study, the authors observed that the combination of finger flexor or quadriceps weakness and endomysial inflammation, and either invasion of non-necrotic muscle fibres or rimmed vacuoles permitted the diagnosis of sIBM with 90 % sensitivity and 96 % specificity [59].

**Table 1:** The ENMC IBM Research Diagnostic Criteria 2011 [83]

Clinical and laboratory features	Classification	Pathological features
Duration >12 months	Clinico-pathologically defined IBM	All of the following:
Age at onset >45 years		Endomysial inflammatory infiltrate
		Rimmed vacuoles
Knee extension weakness ≥hip flexion weakness and/or finger flexion weakness >should abduction weakness		Protein accumulation^a^ or 15–18 nm filaments
CK no greater than 15 × ULN		
Duration >12 months	Clinically defined IBM	One or more, but not all, of:
Age at onset >45 years		Endomysial inflammatory infiltrate
Knee extension weakness ≥hip flexion weakness and finger flexion weakness >should abduction weakness		Up-regulation of MHC-I
		Rimmed vacuoles
		Protein accumulation^a^ or 15–18 nm filaments
CK no greater than 15 × ULN		
Duration >12 months	Probable IBM	One or more, but not all, of:
Age at onset >45 years		Endomysial inflammatory infiltrate
Knee extension weakness ≥hip flexion weakness or finger flexion weakness >should abduction weakness		Up-regulation of MHC-I
		Rimmed vacuoles
		Protein accumulation^a^ or 15–18 nm filaments
CK no greater than 15 × ULN		

^**a**^Demonstration of amyloid or other protein accumulation by established methods (e.g. for amyloid Congo red, crystal violet, thioflavin T/S, for other proteins p62, SMI-31, TDP-43). Current evidence favours p62 in terms of sensitivity and specificity but the literature is limited and further work required

**Fig. 2:**
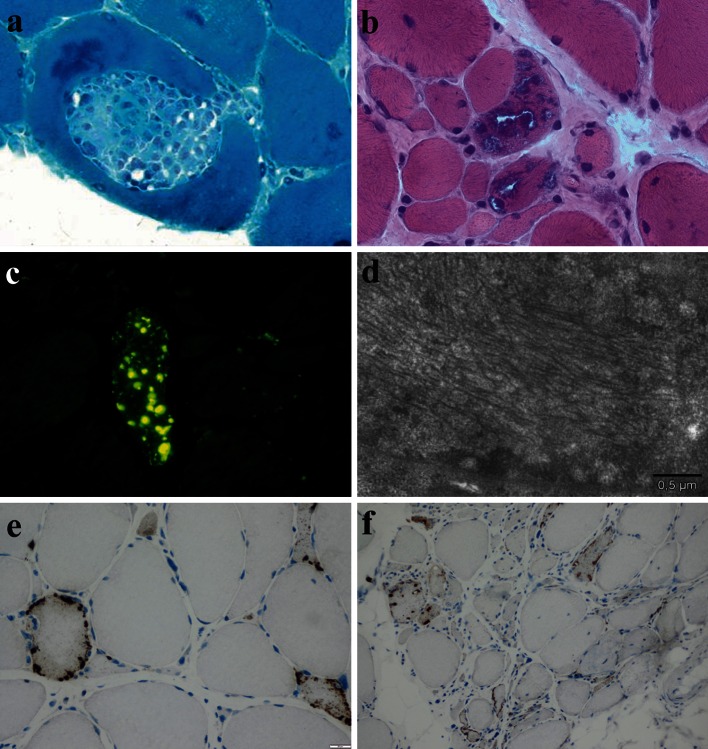
Pathological features of sIBM: **a** partial invasion of a myofiber by inflammatory cells (Gomori trichrome; original magnification ×600). **b** Intracytoplasmic rimmed vacuoles (H&E; original magnification ×400). **c** Evidence of intracytoplasmic and intravacuolar amyloid by staining with pFTAA (original magnification ×400). **d** Intracytoplasmic paired helical filaments illustrated by ultrastructural examination (scale bar represents 0.5 μm). **e**, **f** Immunohistochemical evidence of P62^+^ aggregates in muscle fibres (**e** original magnification ×400, **f** original magnification ×200)

Until recently, the diagnostic gold standard was considered to be certain pathological criteria. These criteria [42] include the presence of inflammatory infiltrates with mononuclear cell invasion of non-necrotic muscle fibres (partial invasion, Fig. 2a), vacuolated muscle fibres (Fig. 2b), and intracellular amyloid protein deposits [detected by fluorescent methods (Congo red, or p-FTAA dye (fluorescent thiophene) [52], Fig. 2c] or 15–18 nm tubulofilaments in the cytoplasm or the nucleus by electron microscopy (Fig. 2d). Electron microscopy is rarely used in everyday practice and the original diagnostic criteria, without formal agreement, have been supplemented with the detection of various proteins by immunohistochemistry [e.g. with antibodies against phosphorylated tau, TDP43, or p62 (Fig. 2 e, f) [22, 32] see below].

Furthermore, it has been noted frequently that the above-mentioned canonical pathological features may be missing in patients with typical clinical features of the condition (leading to a sensitivity of only 11 % [59]), particularly at first presentation, perhaps in part due to a small biopsy not reflecting the whole pathological picture [21, 24, 32, 77]. Repeat biopsy later in the course of the disease may show canonical features absent at presentation, either because they evolve with time (see below), or reflecting sampling error.

Finally, Pestronk [75] proposed classifying sIBM pathologically as an inflammatory myopathy with vacuoles, aggregates and mitochondrial pathology (IM-VAMP); but regarding the clinical features of sIBM, he did not estimate the specificity nor the sensitivity of such pathological criteria (which indeed has been the case with other proposed criteria). Accordingly, mitochondrial abnormalities (COX negative and/or ragged red fibres) and partial invasion have been shown to provide strong evidence for the diagnosis of sIBM [21, 24]. It has been also shown that the increased frequency of ragged red fibres, above the level predicted for normal ageing, is unique to sIBM and not observed in other inflammatory myopathies including polymyositis and dermatomyositis [79].

These diagnostic difficulties relate to the fact that the physiology of sIBM is not yet fully understood and that its proposed mechanisms are still debated [39]. In short, the canonical pathological features facilitated the initial clinical recognition of sIBM as a specific disorder. Additional pathological features have since been recognized (accumulation of various proteins), but no individual pathological feature is diagnostic. The stereotypical clinical features can allow diagnosis even in the absence of the typical histopathologic picture [21].

## Pathogenesis

### Amyloid component of sIBM

Included in the canonical pathological criteria, one of the main features of this disease is the abnormal accumulation of ubiquitinated proteinaceous congophilic inclusions within some muscle fibres. This was first described in 1991 by Mendell et al. [62] who studied the composition of cytoplasmic and intranuclear filamentous inclusions observed by electron microscopy (Fig. 2d) on sIBM muscle biopsies and who observed amyloidogenic birefringent green deposits after Congo red staining. They also observed that the number of amyloid-positive fibres correlated with the number of vacuolated fibres. Perhaps presciently, the authors noted that “the association of amyloid deposits with autophagic vacuoles (i.e. rimmed vacuoles) in IBM raises the likely possibility that the filaments represent a modification of a normal protein within an acidic degradative vacuolar compartment” [62]. Shortly after, Askanas et al. [9, 10] showed by immunohistochemistry that inclusions within vacuolated fibres were immunoreactive with anti-β-amyloid antibodies, and by immunogold electron microscopy that β-amyloid protein immunoreactivity was localized in proximity to cytoplasmic tubulofilaments [9, 10]. The amyloid-β precursor protein (AβPP) overproduction is not yet clarified (for review [12]). AβPP is then cleaved to Aβ40 or Aβ42, and oligomers of these proteins may form subsequently [8]. We will use the term ‘β-amyloid’ throughout the review. These β-amyloid protein deposits are also shown to be accompanied by an increase in plasma Aβ42 protein in sIBM blood samples as compared to polymyositis patients and control subjects [1]. Nevertheless, this increase is also noticed in the blood of dermatomyositis (DM) patients limiting the value of this potential biomarker. Again by immunostaining, Askanas et al. [11] demonstrated the presence of numerous further molecules known to be associated with specific degenerative processes, particularly of the central nervous system such as phosphorylated tau, ubiquitin, α-synuclein and prion protein (for review see [8]). These abnormal protein aggregates are observed within muscle fibres in the form of plaque-like or dotty inclusions [8]. However, protein deposition may not have the same functional implications as in the central nervous system. β-amyloid deposits, for example, are mostly found extracellularly in Alzheimer’s disease while they are intracellular in muscle fibres of sIBM patients.

Before considering further what might be called the ‘amyloid hypothesis’, it must be noted that not all investigators have concluded that the accumulation of proteins associated with neurodegeneration is of specific significance. For example, using a proteomic approach, Parker et al. [73] did not find accumulation of such proteins in sIBM nor did they detect even one peptide from β-amyloid. This lack of detection could be related to limited sensitivity of current proteomic technology [95].

**Fig. 3:**
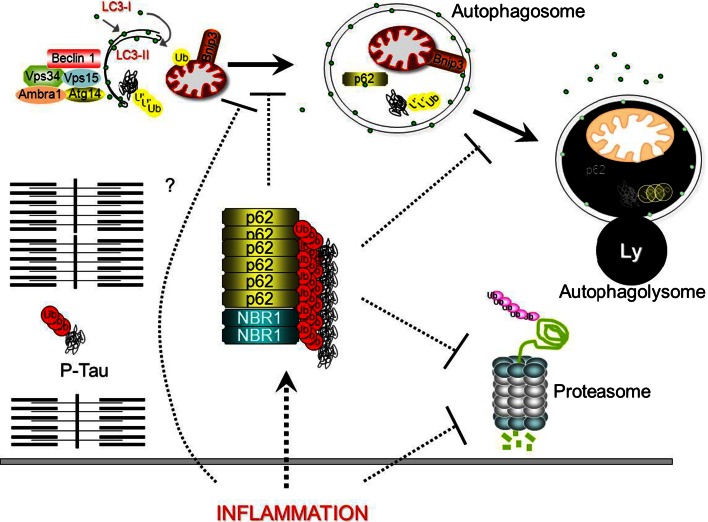
In sIBM, misfolded proteins start to accumulate into aggregates that are positive for P62 and NBR1. The presence of P62-positive aggregates is a consequence of impaired protein clearance of proteasome and autophagy/lysosome (Ly) systems that further impacts on their activities. Overload of the autophagy system with subsequent exhaustion, aspecific absorption and sequestration of the proteasome on the surface of inclusions describe how aggregates negatively affect these systems. Autophagy is characterized by membranes that are committed to growth, thus becoming double membrane vesicles, named autophagosome, that surround a ‘portion’ of cytoplasm, organelles, glycogen and protein aggregates. Autophagy is triggered by the activation of a regulatory complex (containing Vps34, Beclin 1, Vps15, Ambra1, Atg14) that induces LC3 recruitment to the nascent autophagosome (isolation membrane). Selective removal of organelles including mitochondria (mitophagy, a specific form of autophagy) requires several signals. For example in mitophagy, Bnip3 factors are recruited on damaged mitochondria and by binding LC3 allow the recruitment of the vesicle on the surface of the altered mitochondria. Proteins that are committed for lysosomal degradation are labelled by polyubiquitin chains and delivered to the autophagosome by the p62/NBR1 scaffold proteins that bind LC3. Finally, upon autophagosome fusion with lysosome the cargo is destroyed and constituents are recycled by the cell to rebuild organelles/proteins or for energy purposes. Whether inflammation triggers protein misfolding aggregation or blocks the autophagy and proteasome system is still an open issue and will certainly be subject of future studies. *Dotted lines* represent unknown mechanisms (Ub = ubiquitin)

Nevertheless, defenders of the theory of β-amyloid-mediated sIBM myofiber injury have tried to understand the physiology of the amyloid deposits by studying protein degradation pathways (Fig. 3). One of the two pathways for protein degradation is the 26S-proteasome; notably, it is responsible for the degradation of abnormal or damaged proteins. The 26S proteasome can be divided into two sub-complexes, the core particle (20S) and the regulatory particle (19S). The 19S assists in deubiquitination, and unfolds ubiquitinated protein substrates that are subsequently translocated into an enclosed cavity formed by the 20S unit. Here, a variety of catalytic sites degrade the substrate into short peptides that are subsequently broken down to amino acids by peptidases and recycled by the cell. In sIBM muscle biopsies, 26S proteasome subunits (19S and 20S) co-localized by immunodetection with phosphorylated tau, ubiquitin and/or β-amyloid protein deposits [35]. Paradoxically, even though both proteasome subunits were upregulated in sIBM muscle, the three main proteolytic activities (trypsin-like, chymotrypsin-like and peptidyl-glutamyl-peptide hydrolytic) of the 20S were dramatically reduced [35]. This is consistent with the fact that proteasomes can destroy only soluble proteins while aggregates are mainly removed by autophagy [37].

The second pathway of protein degradation is autophagy (Fig. 3). A portion of the cytoplasm, organelles, proteins and protein aggregates are engulfed by a double-layered membrane to form a vesicle that is called an autophagosome. Next, autophagosomes dock and fuse with lysosomes to form autolysosomes where the cargo is degraded by acidic hydrolases. The degradation products are transported to the cytosol where they are utilized to build new organelles/proteins or for energy production [88]. Autophagy can be selective and can remove specifically damaged organelles and/or protein aggregates. Different mechanisms are involved in the selective recognition of the cargo and some are depicted in Fig. 3. Failure of autophagy leads to accumulation of cargo and autophagy substrates such as p62/SQSTM1, NBR1 and tau. Autophagosomes containing amyloid precursor protein and β-amyloid proteins can be observed at increased frequency in muscle fibres of sIBM muscle biopsies, but not in non-myopathic muscle or in non-vacuolated myopathic controls [60]. Moreover, Nogalska et al. [67] observed markers of autophagy induction such as LC3-II in sIBM muscle, but at the same time, a significant decrease of lysosomal enzyme activities of cathepsins D and B, leading to the conclusion that autophagy is also impaired during sIBM. The mechanism explaining why autophagy is impaired, and at which level the autophagy flux is blocked in sIBM, is still not fully understood (see Fig. 3). It is important now to determine whether such failure is secondary to inflammation or is a direct consequence of excessive protein misfolding and aggregation.

Concomitantly p62, a shuttle protein transporting poly-ubiquitinated proteins for both the proteasomal and lysosomal degradation pathways accumulates within muscle fibres into aggregates that are positive for phosphorylated tau [68]. This feature is consistent with the failure of autophagy. Indeed, one of the most important markers of autophagy block is the presence of p62-positive inclusions [89] (Fig. 3). Accumulation of another protein, TDP43 [32, 87], which is one component of the ubiquitinated inclusions in the brain of patients with, for example, frontotemporal dementia, may also relate to proteasome dysfunction [101]. It has been shown that the conditional knock-out of the proteasome (but not of autophagy) in mice induced the accumulation of TDP43 [48]. The presence of p62 and/or TDP43 accumulations, demonstrated by immunostaining, is now recognized as one of the most sensitive pathological findings in sIBM [22, 32]. However, the best diagnostic sensitivity (93 %) and specificity (100 %) were obtained by the combination of characteristic p62 aggregates and increased sarcolemmal and internal major histocompatibility complex class I expression or endomysial T cell infiltrates [22].

Thus, in sIBM, accumulation of aggregates seems to be related to proteasome and autophagy dysfunctions, but with what pathophysiological consequences? Various mouse models have attempted to provide further insight. A double transgenic mouse, overexpressing APP and the *presenilin*-*1* gene under the dependence of a muscle specific promotor, showed increased Aβ42 levels in skeletal muscle and exacerbation of inclusion body myositis-like pathology and motor deficits [50]. Moreover, the overexpression of the proteolytic fragments of mutant (D187N/Y) plasma gelsolin (familial Finnish amyloidosis) also leads to muscle weakness, the appearance of vacuoles and an increase in proteasome and autophagy markers [71]. Specific overexpression of a transgenic heavy chain of major histocompatibility class I (MHC-I) in myofibers leads to a severe myopathy in immunocompetent mice with induction of stress of the endoplasmic reticulum [65, 66]. It was further shown that these changes also occur in immunodeficient mice, indicating that in mice this myopathy may arise without any contribution of the adaptive immune system (i.e. presentation of muscle autoantigens to autoreactive CD8^+^ T cells by MHC-I molecules) [36]. Interestingly in this report, muscle of sIBM patients presented similar proteomic features of endoplasmic reticulum stress in a fashion that was dependent on the level of intracellular accumulation of MHC-I molecules [36]. It is likely that, when the protein degradation systems are overloaded (e.g. here by the MHC-I overexpression), proteins are misfolded and/or ubiquitinated and their accumulation causes a vacuolar myopathy [36]. This situation is also observed in patients with a form of hereditary inclusion body myopathy (hIBM) due to p97/VCP mutations, which is another molecular complex responsible for the regulation of protein degradation through the proteasome and autophagy [49]. Here again, P62 and TDP-43 accumulate in the cytoplasm of p97/VCP mutant-expressing cells and transgenic mouse muscle [48]. Furthermore in sIBM, the β-amyloid β42 deposits co-localized with dysferlin, which is absent from the sIBM muscle fibre sarcolemma (contrary to normal muscle where dysferlin is localized at the sarcolemma) [23]. Knowing that this protein is involved in the sarcolemmal repair of muscle fibres [16], this interaction may aggravate the myopathy.

### Inflammatory component of sIBM

**Fig. 4:**
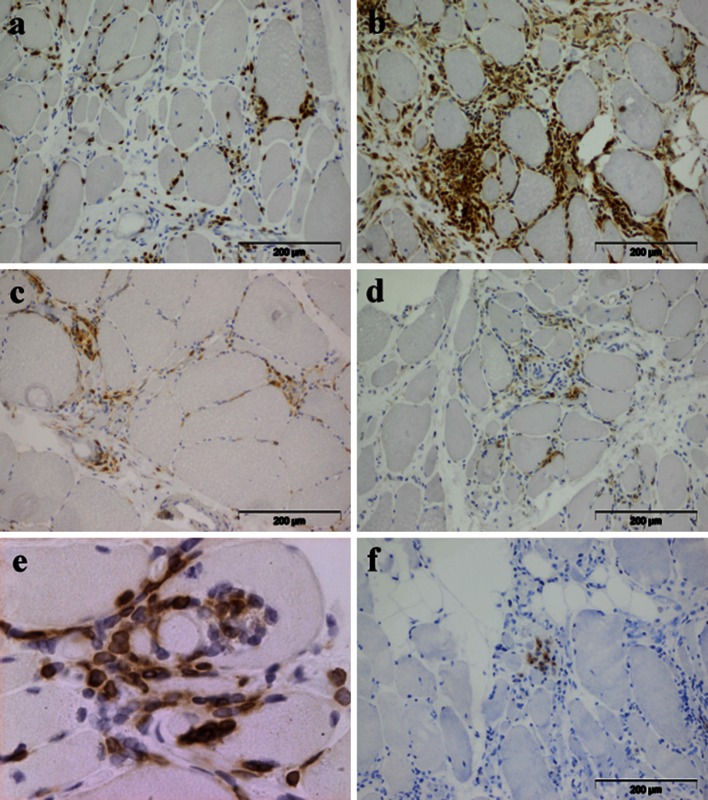
Composition of the inflammatory infiltrate in sIBM muscle: **a** CD8^+^ cells. **b** CD68^+^ macrophages. **c** CD4^+^ cells. **d** CD20^+^ B cells. **e** Clonally Vβ1 expanded cells. **f** CD138^+^ plasma cells

The second pathological hallmark of sIBM is the presence of inflammatory infiltrates (Fig. 4). These infiltrates are rich in lymphocytes (mostly CD8^+^ T cells, Fig. 4a) and macrophages (Fig. 4b), while CD4^+^ T cells (Fig. 4c) and B lymphocytes (Fig. 4d) are less abundant. CD8^+^ T cell- and macrophage-rich infiltrates are regularly observed invading non-necrotic fibres (Fig. 2a) [7]. Invading CD8^+^ T cells express co-stimulatory molecules such as inducible T cell co-stimulator (ICOS) which may promote lymphocyte activation by providing secondary signals to T cells in addition to T cell receptor stimulation by antigen, and elicit cytotoxic function markers such as perforin [93]. CD8^+^ T cells can be expanded ex vivo from muscle of polymyositis and sIBM patients. These clones show cytotoxic activity against autologous myotubes in vitro [46]. We have previously demonstrated shared oligoclonal expansions of CD8^+^ cells in both peripheral blood and muscle inflammatory infiltrates of sIBM patients (Fig. 4e) [31]. These clones persist in repeated muscle biopsies [4], as they do in polymyositis [18]. Finally, these expanded cytotoxic CD8^+^ T cells become CD28^null^ [2, 72], a phenotype of chronically activated and terminally differentiated T cells. To develop into functional cytotoxic effectors, CD8^+^ T cells must recognize, via their T cell receptor, specific antigenic epitopes presented by the target cells in a MHC-I-restricted manner. Not surprisingly then, diffuse overexpression of MHC-I is observed on the surface of myofibers in sIBM [33] and is even more prominent in sIBM compared to other forms of myositis [74], allowing the presentation of (today still unknown) antigens to effector T cells. The attack of myofibers by cytotoxic T cells seems to be related to local inflammation associated with the induction of the interferon gamma receptor, up-regulation of several interferon gamma-induced genes [47] and presence of several cytokines such as IFN-γ, IL-1β, TNF-α [70] and tumour necrosis factor-like weak inducer of apoptosis (TWEAK) [64].

On the other hand, immuno-regulatory second signals such as programmed death ligand 1 (PD-L1, formerly referred to as B7-H1) are also triggered, presumably as a negative feedback loop. PD-L1 is expressed in polymyositis, dermatomyositis and sIBM muscle fibres but not in normal muscle. Staining is predominantly localized (in muscle fibres as well as mononuclear cells) to areas of strong inflammation [102]. Furthermore, cultured human myoblasts express high levels of PD-L1 after stimulation with the inflammatory cytokine IFN-γ [102]. Another mechanism of control of the specific immune responses relates to regulatory T cells (Treg). Congenital or acquired Treg deficiency causes multi-organ autoimmune disease in mice and in humans [20, 55, 98, 103]. We were the first to describe a Treg deficiency (but with normal function) in the peripheral blood of sIBM patients [2]. Indeed, we observed in 22 sIBM patients, compared to 22 sex- and age-matched healthy subjects, that sIBM patients had a decreased frequency of circulating regulatory T cells (CD4^+^CD25^+^CD127^low^FOXP3^+^, 6.9 ± 1.7 %; vs. 5.2 ± 1.1 %, *p* = 0.01) that displayed normal suppressor function [2].

Also recently, new arguments, coming from microarray analyses, have shown that it is not only cellular responses that are involved in sIBM but also humoral responses [41]. In contrast to normal muscle, immunoglobulin genes are the most abundant transcripts in sIBM, and antibody-secreting plasma cells (CD138^+^) are frequently observed (Fig. 4f) in higher numbers than B cells which remain rather rare (Fig. 4d) [41]. Recently, Ray et al. [78] isolated single plasma cells by microdissection directly from IBM-derived muscle tissue sections and analysed a series of recombinant immunoglobulins produced from these cells; the muscle molecular target of these antibodies appeared to be desmin. Of note, anti-desmin antibodies have been described in different autoimmune conditions (such as lupus and autoimmune thrombocytopenic purpura [94, 97]) and might not be of any specific significance, but in inherited desminopathies, clinic-pathological features include distal involvement and rimmed vacuoles with amorphous deposits [38]. These desmin aggregates co-localized with ubiquitinated proteins (and notably mutant ubiquitin which is resistant to proteasome degradation) and p62 [69]. It is therefore questionable whether those intramuscular secreted anti-desmin antibodies participate in proteasome dysfunction.

Despite the clear evidence of global immune activation in sIBM, the question remains as to whether sIBM is primarily an inflammatory or a degenerative myopathy. If amyloid is the consequence of a primary immune reaction, including secretion of cytokines that increase MHC-I expression to such an extent that protein degradation capabilities are overloaded [36], then targeted immune intervention (e.g. biotherapies) at an early stage of the disease may be useful. On the contrary, if sIBM is primarily a degenerative disease in which accumulation of unfolded proteins causes a secondary immune reaction, immunointervention may be of limited, if any, benefit. Thus, we will review below the pros and the cons for both hypotheses.

## Arguments for degeneration as initial event/trigger

The main argument comes from the lack of significant beneficial clinical effect from conventional immunosuppressant drug therapy. Eight prospective double-blind studies have shown that conventional immunosuppressants (corticosteroids or methotrexate) or immunomodulatory regimens (polyvalent immunoglobulins, beta interferon, oxandrolone) have minimal or no benefit for sIBM patients [14, 28, 30, 43, 44, 57, 84, 100]. It has to be noted that the limited number of validated and sensitive disease activity and damage assessment tools specific for sIBM [45] are clearly a limitation in evaluating the clinical trial data, and probably all of these studies were underpowered, in terms of numbers and duration. Thus, we cannot be absolutely certain of complete lack of response to such treatments, although our own observations strongly support the lack of clinical efficacy. We undertook a long-term observational study of 136 sIBM patients of whom 71 patients (52 %) received immunosuppressive treatment [prednisone in 92 %, associated, in 65 %, with other immunomodulatory drugs (intravenous immunoglobulins, methotrexate or azathioprine)] for a median duration of 3.5 years [17]. At the last assessment, treated patients had not only not improved, but were more severely affected, based on disability scales (Walton *p* = 0.007, RMI *p* = 0.004) and on the sIBM weakness composite index (*p* = 0.04), than those who were not treated. The first stage of disease progression, towards handicap in walking, also occurred more rapidly among patients receiving immunosuppressive treatments (HR = 2.0, *p* = 0.002). We therefore concluded that immunosuppressive treatments do not ameliorate, and may even aggravate, the natural course of sIBM [17]. We have to note that there are other presumed autoimmune conditions that show similar resistance to immunosuppressant therapy, notably multiple sclerosis (in its primary progressive form) and scleroderma. The immunosuppressant resistance may then not be a definite argument against the autoimmune nature of sIBM.

## Arguments for inflammation as initial event/trigger

sIBM has been frequently described in association with other autoimmune disorders [15, 24, 53]. For instance, in a Dutch study of 52 sIBM patients, 17 patients (33 %) had associated autoimmune disorders including autoimmune thyroiditis (*n* = 6), rheumatoid arthritis (*n* = 4) and Sjögren syndrome (*n* = 4), with 3 patients with multiple autoimmune disorders [15]. The prevalence of autoantibodies is also increased in sIBM [24, 80]. For instance, in an Australian study, 51 sIBM patients were compared to 198 controls and the frequency of positive antinuclear autoantibodies was higher in sIBM (29.4 vs. 8.6 %, *p* < 0.001), and among antibodies against extractable nuclear antigens (in order of their frequency), anti-SSA/Ro-52 or Ro-60, SSB/La, RNP-A or C, and PM-Scl75 were statistically more frequently observed in sIBM patients [80].

Recently, apparently sIBM-specific autoantibodies have been described. An antibody recognizing a 43 kD antigenic target was found in the serum of 13 out of 25 sIBM patients but not in 40 controls (healthy subjects or patients with other inflammatory myopathies) [86]. The antigenic target of this sIBM associated autoantibody has been identified by two independent teams as cytosolic 5′-nucleotidase 1A [56, 76] an enzyme involved in nucleotide metabolism which is expressed at a relatively high level in skeletal muscle. Anti-NT5C1A autoantibodies were present in 33 % [76] and 34 % [56] of sIBM patients, as compared to ≤5 % in polymyositis and dermatomyositis [56, 76]. Nonetheless, the specificity of this new anti-NT5C1A autoantibody for the diagnosis of sIBM has not yet been fully evaluated and further studies are required, particularly in other groups of connective tissue diseases such as rheumatoid arthritis, lupus and systemic sclerosis. To date, our unpublished data suggests that this autoantibody can also be present in rheumatologic disorders such as lupus and Sjögren syndrome, so its specificity may not be as high as initial reports, in a more restricted group of patients, suggested.

Evidence for a sIBM-prone immunogenetic background comes from observations of a strong association with prominent alleles of HLA class I or II (such as alleles B8 and notably DRbeta1*0301) [15, 53, 81], a situation not observed in hereditary inclusion body myopathies [53].

From a pathological point of view, Pruitt et al. [77] have shown that in muscle biopsies derived from 31 patients, the frequency of invaded fibres (mean 24.3/1,000) was several fold higher than that of congo red positive fibres (mean 3.1/1,000) or that of necrotic fibres (mean 3.1/1,000), and they concluded on the importance of an immune-mediated mechanism in sIBM. Sarcolemmal HLA class I expression, which is absent in normal muscle [6], is upregulated in myositis in general [27] and in sIBM in particular [74]. The immunohistological detection of HLA class I on the sarcolemma is thus considered to be a marker of myositis. In sIBM, the prevalence of HLA staining on muscle fibres is higher compared to dermatomyositis and polymyositis: expression of HLA class I was found in 67 % of muscle biopsies from patients with dermatomyositis, in 61 % with polymyositis, and in 96 % with sIBM (on 208 analysed biopsies) [74].

Finally, and in contrast to the observations noted above concerning the outcome of prospective, double-blind, studies of immunosuppressive therapies in sIBM, two clinical trials, which included only a small number of patients, suggested that massive immunosuppression can lead to strength stabilization. The first compared six patients treated with anti-T lymphocyte globulin and methotrexate, with five patients treated with methotrexate only. The latter showed a significant loss of strength after 12 months but the anti-T lymphocyte globulin group remained stable [58]. In the second study, 13 patients were evaluated by quantitative muscle strength testing to determine their natural history over 1 year. These patients were then treated by alemtuzumab (Campath™) and showed a transient stabilization of strength after 6 months [29]. Even if the results of the latter study have been debated [40], both studies suggest the possible involvement of adaptive immune responses in disease progression.

As indicated above, sIBM is accompanied by degenerative protein accumulations, and deficient protein degradation pathways (both proteasomal and lysosomal) and one accumulated protein candidate might be HLA class I [36]. This situation exists in patients with a hereditary inclusion body myopathy due to p97/VCP mutation, which is a complex responsible for the regulation of protein degradation through these two pathways, but this hereditary inclusion body myopathy is not accompanied by significant inflammation [49], neither the animal model [48]. In the same vein, the other classical hereditary inclusion body myopathy is caused by UDP-*N*-acetylglucosamine-2-epimerase/*N*-acetylmannosamine kinase (*GNE*) gene mutation (leading to quadriceps-sparing myopathy). *GNE* encodes for a key enzyme in sialic acid biosynthesis and the myopathy is associated with the formation of autophagic vacuoles [61]. But this vacuolar myopathy is essentially not accompanied by muscle inflammation [25], except in three cases, from two families [5, 54]. Nevertheless and despite the general absence of inflammation, iNOS and αB-crystallin, two markers of cell stress, are present in normal appearing fibres of *GNE* patients and correlated with pro-inflammatory markers (such as IL-6), which remained expressed at levels comparable to control muscles [34]. Furthermore, mouse models of *GNE* do not show features of inflammation [105]. Experimentally, a transgenic mouse model of Finnish-type familial amyloidosis associated with gelsolin amyloidosis in skeletal muscle (with the gelsolin transgene under the control of a muscle specific promoter), is associated with amyloid deposits with foci of mononuclear cell infiltration [71]. In a model with genetically augmented Aβ42 levels in skeletal muscle, there was no inflammation [50]. Hence, degenerative processes in muscle induce cell stress but do not clearly and unequivocally elicit inflammation.

## Influence of inflammation on amyloid deposits and/or muscle atrophy

In a sIBM transgenic mouse model, that is marked by enhanced levels of Aβ1-42 in skeletal muscle (of note, with no obvious muscle inflammatory infiltrates) [50], the long-term influence of inflammation, induced by chronic lipopolysaccharide injections, included increased Aβ generation and enhanced tau phosphorylation [51].

In muscle biopsies of sIBM patients, compared to other forms of myositis, Schmidt et al. [90] found that expression of the mRNA of β-amyloid precursor protein significantly and consistently correlated with inflammation and enhanced mRNA levels of chemokines (CXCL-9, CCL-4 and CCL-3) and IFN-γ. To assess the influence of inflammation on β-amyloid accumulation, these authors performed in vitro experiments in human myotubes, and showed that exposure to IL-1β caused up-regulation of APP with subsequent intracellular aggregation of β-amyloid [90]. Furthermore, exposure to interleukin-1β, in combination with interferon-γ induced intracellular production of nitric oxide, was associated with necrotic cell death in muscle cells [91]. Hence, inflammation in muscle can elicit amyloid accumulation. On the other hand, by studying biopsies of sIBM patients before and after prednisone ± polyvalent immunoglobulins, the same group [106] showed that these treatments decrease inflammation in muscle, but do not significantly suppress myotoxic and cell stress mediators such as nitric oxide.

As we have seen previously, various pro-inflammatory cytokines are secreted in the milieu of sIBM inflammatory infiltrates, notably TWEAK [64]. TWEAK induces its effect through its receptor, the fibroblast growth factor-inducible gene 14 receptor (Fn14), on neighbouring cells [104]. In sIBM muscle biopsies (but not in dermatomyositis nor in polymyositis) a dysregulation of the TWEAK-Fn14 axis is observed, with an increase of its expression and secretion, which inhibited (in a reversible manner) the myogenic differentiation of mesoangioblasts [64]. TWEAK appeared then as a powerful muscle-wasting cytokine, inducing progressive muscle fibre atrophy, and simultaneously as a negative regulator of regenerative myogenesis [64].

Moreover, Rigyel et al. [85] have shown a correlation between the number of invading CD3-positive cells (lymphocytes) or CD68-positive cells (macrophages) and COX-deficient muscle fibres (respectively, *p* = 0.007 and 0.04). These correlations between the degree of inflammation and signs of mitochondrial dysfunction strongly suggest a causative link between them. Furthermore, evidence of mitochondrial dysfunction is also highly correlated with fibre atrophy (*p* < 0.0001) [85]. The authors concluded upon “a role for inflammatory cells in the initiation of mitochondrial DNA damage, which when accumulated causes respiratory dysfunction, fibre atrophy and ultimately degeneration of muscle fibres” [85].

## In conclusion: the chicken and the egg: which came first?

From the various arguments summarized above, a possible scenario for the pathogenesis of sIBM, and one that we favour, is that it may start with inflammation within muscle (of unknown aetiology but postulates include viral infections and muscle micro-trauma by eccentric exercise [99]) with the histological features long considered the hallmark of polymyositis (inflammatory cells, invasion of non-necrotic muscle fibres, necrotic and regenerating muscle fibres, but without vacuoles or amyloid). Interestingly, pure polymyositis (i.e. without overlap clinical signs and/or without myositis specific autoantibodies) appears, using current criteria, to be very rare [96], being described as an over-diagnosed entity [63] and even as a mythological beast [3].

From a clinico-pathological standpoint, Chahin and Engel [24] showed that in 107 patients whose biopsies were initially read as polymyositis or sIBM, they were able to distinguish, by combining biopsy and clinical criteria, a third group, which they called PM/IBM, with a biopsy diagnosis of polymyositis but with clinical features of sIBM. Arguably, this third group only exists because of adherence to earlier diagnostic criteria, and can be understood better if it is accepted that patients with sIBM may not show the currently considered canonical pathological features at an early stage of the disorder. Most of the apparently pure polymyositis cases might be, or become, sIBM, representing an intermediate phase falling into the PM/IBM category [24]. In the same vein, Brady et al. [21] have shown in a series of 67 sIBM patients that the presence of rimmed vacuoles on muscle biopsy was more common in older patients (74 vs. 66 years; *p* = 0.04), suggesting that rimmed vacuoles may be a later feature of the disease. Moreover, no differences in disease characteristic or progression were observed between patients with or without vacuoles [21].

**Fig. 5:**
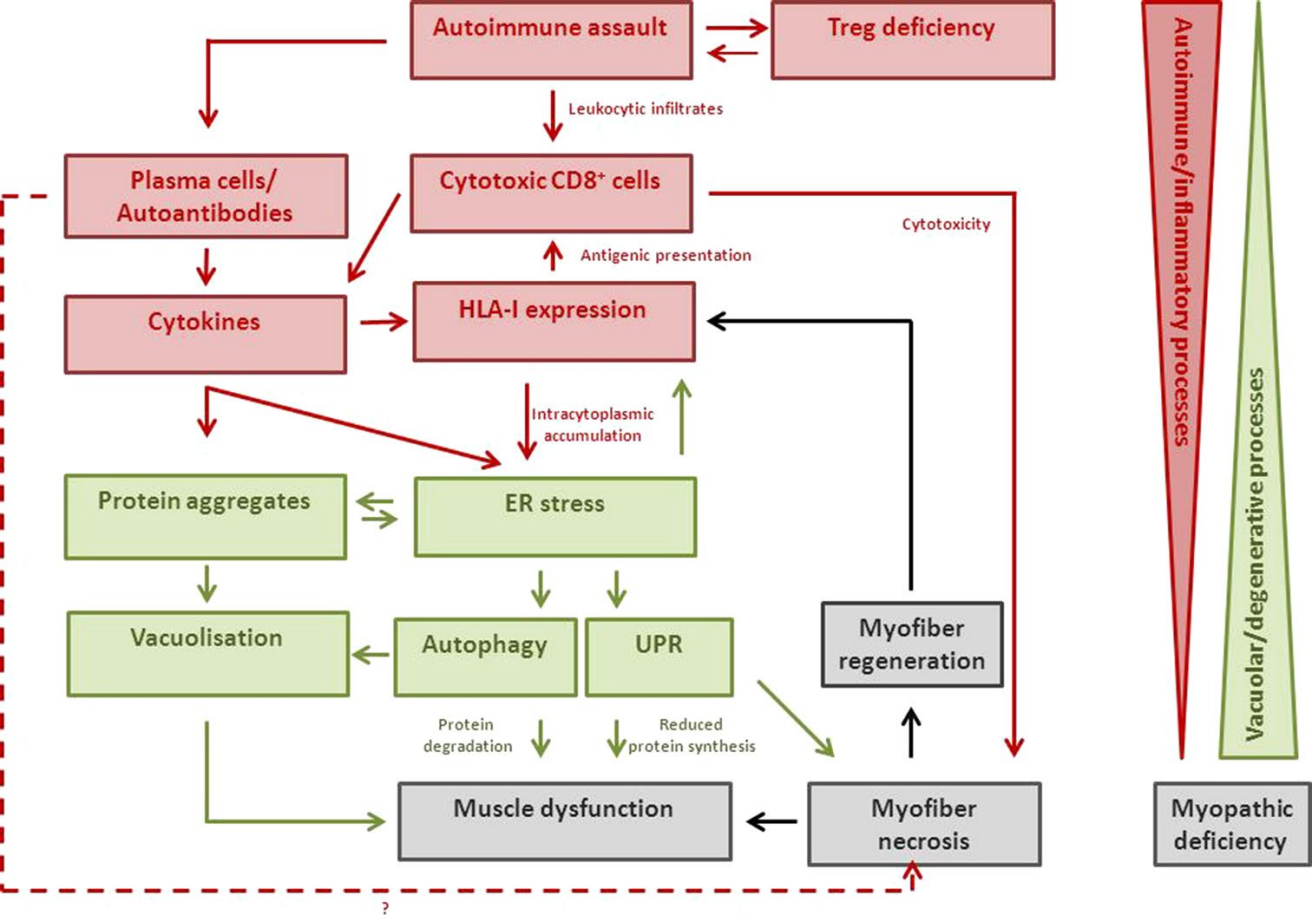
A possible scenario for the pathogenesis of sIBM is that it may start with inflammation within muscle (the different actors are represented in *red*). The rush of leukocytes attracted by chemokines and cytokines may induce fibre injury and HLA-I overexpression. If the protein degradation systems are overloaded (due to genetic predisposition, particular HLA-I subtypes or ageing), amyloid and other protein deposits (represented in *green*) may appear within muscle fibres, reinforcing the myopathy in a vicious circle. The opposite scenario where amyloid deposits come first leading to a secondary inflammatory reaction is less probable since (apart some rare exceptions) neither hereditary inclusion body myopathy nor animal models of forced amyloid deposits (with proteasomal and/or autophagosomal pathway deficiencies) are accompanied by inflammation

The rush of leukocytes, attracted by chemokines and cytokines, may induce fibre injury, mitochondrial dysfunction and HLA class I overexpression through, presumably, components of pro-inflammatory cell stress mechanisms such as nitric oxide production [92]. If the protein degradation systems are overloaded (perhaps failing to cope because of genetic predisposition, particular HLA class I subtypes or ageing), amyloid and other protein deposits may appear within muscle fibres, reinforcing the myopathy in a vicious circle, which is clinically manifest as progressive muscle weakness. Figure 5 tries to summarize these hypothetical physiological pathways starting from inflammation: the egg, if we accept that it came first! The opposite scenario, where amyloid deposits come first, leading to a secondary inflammatory reaction, is in our opinion, less probable since (apart some rare exceptions) neither hereditary inclusion body myopathy nor animal models of forced amyloid deposits (with proteasomal and/or autophagosomal pathway deficiencies) are accompanied by inflammation.
